# Cr-Doped Li_2_ZnTi_3_O_8_ as a High Performance Anode Material for Lithium-Ion Batteries

**DOI:** 10.3389/fchem.2020.600204

**Published:** 2021-01-28

**Authors:** Xianguang Zeng, Jing Peng, Huafeng Zhu, Yong Gong, Xi Huang

**Affiliations:** ^1^Institute of Material, Sichuan University of Science and Engineering, Zigong, China; ^2^Material Corrosion and Protection Key Laboratory of Sichuan Province, Zigong, China; ^3^Langxingda Technology Co, Ltd., Zigong, China

**Keywords:** lithium ion battery, Li_2_ZnTi_3_O_8_, Cr doping, anode material, Li_2_ZnTi_2.9_Cr_0.1_O_8_

## Abstract

Li_2_ZnTi_2.9_Cr_0.1_O_8_ and Li_2_ZnTi_3_O_8_ were synthesized by the liquid phase method and then studied comparatively using X-ray diffraction (XRD), scanning electron microscopy (SEM), X-ray photoelectron spectroscopy (XPS), galvanostatic charge–discharge testing, cyclic stability testing, rate performance testing, and electrochemical impedance spectroscopy (EIS). The results showed that Cr-doped Li_2_ZnTi_3_O_8_ exhibited much improved cycle performance and rate performance compared with Li_2_ZnTi_3_O_8_. Li_2_ZnTi_2.9_Cr_0.1_O_8_ exhibited a discharge ability of 156.7 and 107.5 mA h g^−1^ at current densities of 2 and 5 A g^−1^, respectively. In addition, even at a current density of 1 A g^−1^, a reversible capacity of 162.2 mA h g^−1^ was maintained after 200 cycles. The improved electrochemical properties of Li_2_ZnTi_2.9_Cr_0.1_O_8_ are due to its increased electrical conductivity.

## Introduction

Lithium-ion batteries (LIBs) are a new type of rechargeable batteries that are characterized by high specific capacities and specific energies, small volumes, long life cycles, low cost, low energy consumption, low self-discharge efficiencies, small internal resistance, and high working voltages (Yoshio, 2001; Junmin et al., 2005; Dong-il and Han, 2006; Wang M. X. et al., 2019; Wang S. et al., 2019; Huang et al., 2020; Wang et al., 2020; Yi et al., 2020a), and they have wide applications, including in mobile phones, laptops, cameras, digital cameras, electric automobiles, energy storage, aerospace, and space exploration (Tarascon and Armand, 2001; Xiao et al., 2018, 2019; An et al., 2019; Hong et al., 2019). The selection of suitable anode materials for LIBs is extremely important for the performance of these batteries to have excellent life cycles and charge/discharge rate characteristics.

Recently, there have been numerous studies of anode materials, including LiTi_2_O_4_, Li_2_Ti_3_O_7_, Li_2_Ti_6_O_13_, Li_4_Ti_5_O_12_, Na_2_Li_2_Ti_6_O_14_, TiNb_2_O_7_, and Li_2_ZnTi_3_O_8_ (Tang et al., 2014a; Chen B. K. et al., 2015; Li G. H. et al., 2016; Liu et al., 2016; Li Z. F. et al., 2016; Yi et al., 2020b). Li_2_ZnTi_3_O_8_ with the cubic spinel structure has been considered as a promising material because of its lack of toxicity, low cost, relatively high theoretical capacity of 227 mA h g^−1^, and its low discharge voltage plateau of ~0.5 V (vs. Li/Li^+^) (Jović et al., 2009; Chen B. K. et al., 2015; Chen W. et al., 2015). However, compared with other anode materials, Li_2_ZnTi_3_O_8_ suffers from low electronic conductivity and an even worse rate performance, which means that its performance in practice is lower than the theoretical value (Tang and Tang, 2014; Chen B. K. et al., 2015). Hence, by increasing its electronic conductivity, its use can be extended to a wider range of applications.

Until now, this problem has been solved by applying a synthesized coating containing conductive species, nano-sized particles, and doping ions (Shenouda and Murali, 2008; Qi et al., 2009; Tian et al., 2010; Lin et al., 2011, 2013; Yu et al., 2011; Zhang et al., 2011; Bai et al., 2012; Jhan and Duh, 2012; Wu et al., 2013; Xu et al., 2013; Mani et al., 2014; Tang et al., 2014a; Yi et al., 2020c). As is widely known, by using a synthesized coating with conductive species on Li_2_ZnTi_3_O_8_, the transportation of electrons can be improved while decreasing the particle size, which further accelerates ionic transportation. Moreover, it has been shown that doping ions into the material can increase its internal electronic conductivity (Lee et al., 2010; Fang et al., 2013; Lin et al., 2014). According to previous studies, the introduction of Al (Tang et al., 2014b), Ag (Tang et al., 2014a), Mo (Wang M. X. et al., 2019), and Ti (III) (Chen et al., 2018) to Li_2_ZnTi_3_O_8_ greatly improved the electrochemical properties of these materials. For example, Tang et al. (2014b) partially replaced Ti with Al, and the resulting Li_2_ZnTi_2.9_Al_0.1_O_8_ composite showed a given capacity of 223.1 mA h g^−1^ at 0.1 A g^−1^ (Tang et al., 2014b). Wang M. X. et al. (2019) improved the electronic conductivity of the Li_2_ZnTi_3_O_8_ compound by introducing Mo, and Li_2_Zn_0.93_Mo_0.07_Ti_3_O_8_@graphene showed a high cycle capacity at high current densities of 5 A g^−1^, with a power rating of 153 mA h g^−1^ still being delivered on the 200th cycle (Wang M. X. et al., 2019). Recently, researchers have paid close attention to the use of Cr doping, which, according to one study (Ruan et al., 2017), can further enhance the electronic conductivity. Pan et al. (2018) have successfully prepared Cr-doped γ-Fe_2_O_3_/rGO cathode material by microwave method. The as-obtained 4.0 at% Cr-doped γ-Fe_2_O_3_/rGO sample exhibits the capacity of 1,060 mAh g^−1^ after 100 cycles at 100 mA g^−1^. Liu et al. (2018) have prepared Fe_1.95_Cr_0.05_F_5_·H_2_O material, which retains a discharge capacity of 171 mAh g^−1^ after 100 cycles at 0.1 C (1 C = 200 mAh g^−1^). Feng et al. (2009) have successfully synthesis Cr-LiV_3_O_8_ cathode material, and it showed an excellent electrochemical performance, with the retention of 94.4% after 100 cycles. To the best of our knowledge, the introduction of Cr^3+^-doped ions into Li_2_ZnTi_3_O_8_ has not yet been reported. In this paper, we describe the improvement in electrochemical performance of Li_2_ZnTi_3_O_8_ anode material as a result of Cr doping.

## Materials and Methods

Li_2_ZnTi_2.9_Cr_0.1_O_8_ was prepared by the liquid phase method. Stoichiometric amounts of TiO_2_, (CH_3_COO)Li, (CH_3_COO)_2_Zn, and Cr(NO_3_)_3_ were mixed with anhydrous ethanol as solvent. The mixture was dried at 80°C for 2 h and then roasted in a muffle furnace at 700°C for 10 h to obtain the final Li_2_ZnTi_2.9_Cr_0.1_O_8_ compound. For comparison, Li_2_ZnTi_3_O_8_ was also synthesized without using Cr(NO_3_)_3_ as the raw material.

X-ray diffraction (XRD, Brook AXS's D2 PHASER) was used to examine the crystalline phase of both Li_2_ZnTi_3_O_8_ and Li_2_ZnTi_2.9_Cr_0.1_O_8_, which was recorded within the range of 10–70° (2θ). Scanning electron microscopy (SEM, TESCAN VEGA3) was used to examine the morphology of the samples. X-ray photoelectron spectroscopy (XPS) with Cr-Kα radiation was used to monitor the surface electronic states of the elements that were the sources of X-rays.

To construct the working electrodes, the as-prepared material, Super-P and LA-132 were mixed in weight ratios of 80:10:10 with water as solvent, and the prepared slurry was then pasted onto copper foil and dried at 100°C for 10 h in a vacuum oven. The working electrode, lithium metal, Celegard 2400 separator, and 1 M LiPF_6_ electrolyte solution containing a 1:1:1 mixture of ethylene carbonate (EC), dimethyl carbonate (DMC), and ethylmethyl carbonate (EMC) were used to prepare CR2032 coin cells in an Ar-filled glovebox. All electrochemical performances were tested in the voltage range of 0.05–3.0V at room temperature.

## Results and Discussion

**Figure 1:**
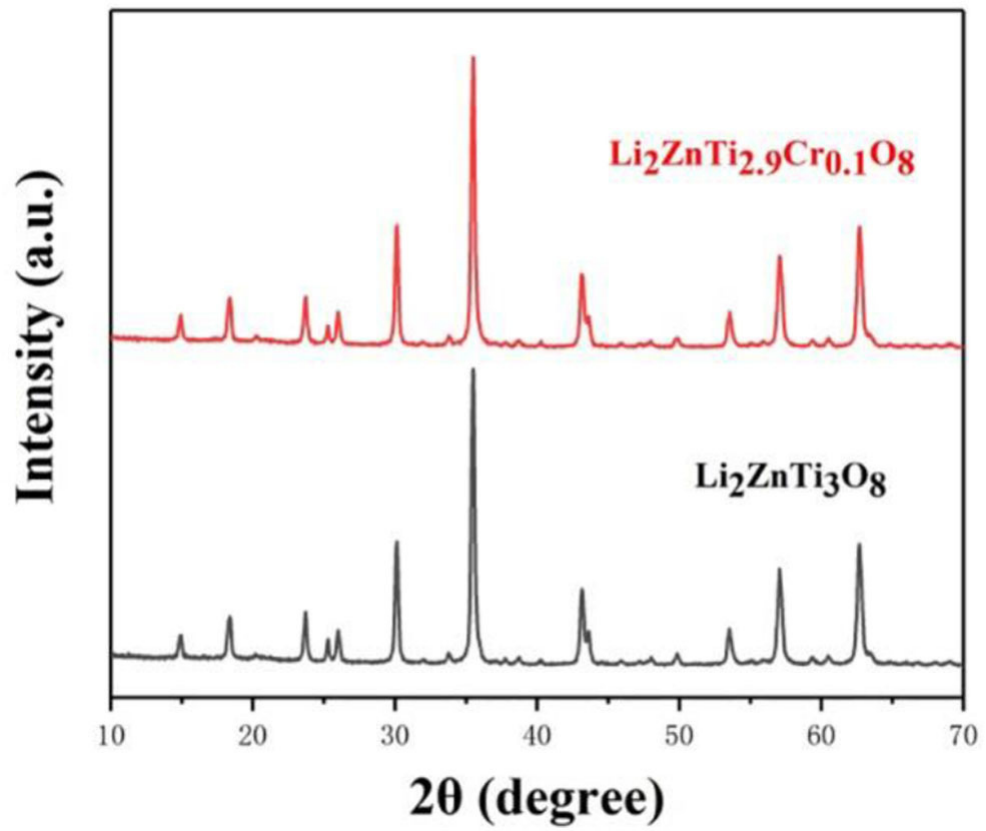
XRD patterns of Li_2_ZnTi_3_O_8_ and Li_2_ZnTi_2.9_Cr_0.1_O_8_.

Figure 1 shows the XRD patterns of Li_2_ZnTi_3_O_8_ and Li_2_ZnTi_2.9_Cr_0.1_O_8_. It is clear from the figure that the diffraction peaks of the samples conform to the standard diffraction peaks of cubic spinel Li_2_ZnTi_3_O_8_, which demonstrates that the presence of Cr(NO_3_)_3_ does not obviously affect the structure of cubic spinel Li_2_ZnTi_3_O_8_. The crystal lattice constants calculated from the recorded XRD data are listed in Table 1, and the lattice parameters of Li_2_ZnTi_3_O_8_ and Li_2_ZnTi_2.9_Cr_0.1_O_8_ were estimated to be a = 8.4305 and 8.4340 Å, respectively. The lattice parameters increased following introduction of Cr^3+^ into the Li_2_ZnTi_3_O_8_ crystal structure due to the larger radius of Cr^3+^ (0.062 nm) compared with that of Ti^4+^ (0.061 nm) (Novikova et al., 2018), which promotes the transportation of lithium ions and further enhances the electrochemical performance (Tai et al., 2020).

**Table 1:** The crystal lattice constant for Li_2_ZnTi_3_O_8_ and Li_2_ZnTi_2.9_Cr_0.1_O_8_.

**Samples**	**Lattice parameters**	
	**a(Å)**	**V(Å^**3**^)**
Li_2_ZnTi_2.9_Cr_0.1_O_8_	8.4340	599.9303
Li_2_ZnTi_3_O_8_	8.4305	599.1837

**Figure 2:**
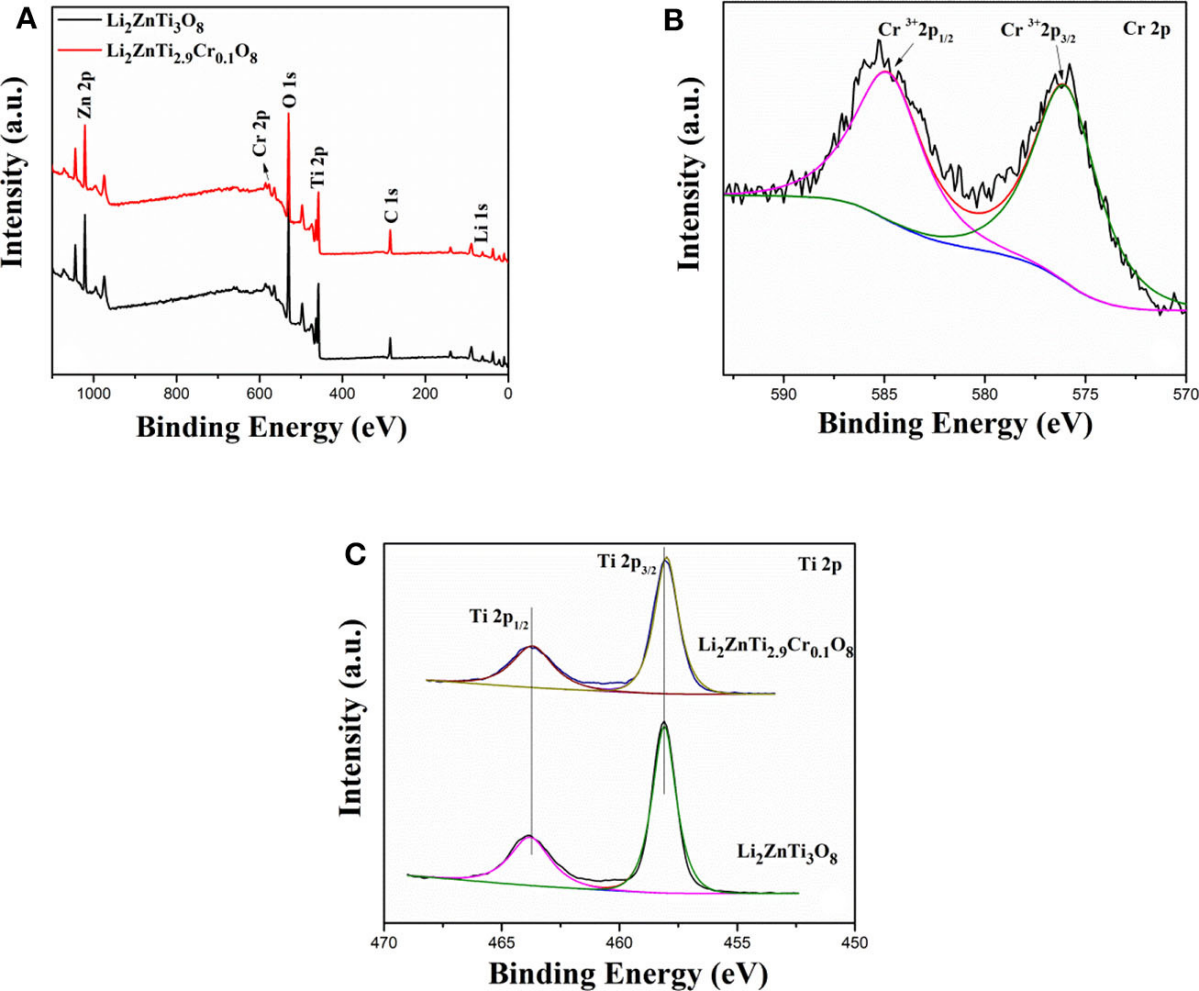
XPS spectra of **(A)** XPS survey spectrum, **(B)** Cr 2p, **(C)** Ti 2p of Li_2_ZnTi_3_O_8_ and Li_2_ZnTi_2.9_Cr_0.1_O_8_.

Additional information about the surface electronic states of the elements was gained from XPS measurements. Figure 2A shows the XPS survey spectra of Li_2_ZnTi_2.9_Cr_0.1_O_8_. As can be seen from the figure, the peaks of Li 1s, C 1s, O 1s, Ti 2p, Zn 2p, and Cr 2p are clearly visible. Figure 2B reveals that two peaks centered at approximately 576.36 and 585.60 eV correspond well to the Cr 2p_3/2_ and Cr 2p_1/2_ peaks, demonstrating that Cr is present as Cr^3+^ ions in the as-synthesized Li_2_ZnTi_2.9_Cr_0.1_O_8_. The two peaks shown in Figure 2C centered at approximately 458.4 and 464.40 eV are detected for Li_2_ZnTi_3_O_8_ and Li_2_ZnTi_2.9_Cr_0.1_O_8_, which can correspond to the peaks of Ti (IV) 2p_3/2_ and Ti (IV) 2p_1/2._

**Figure 3:**
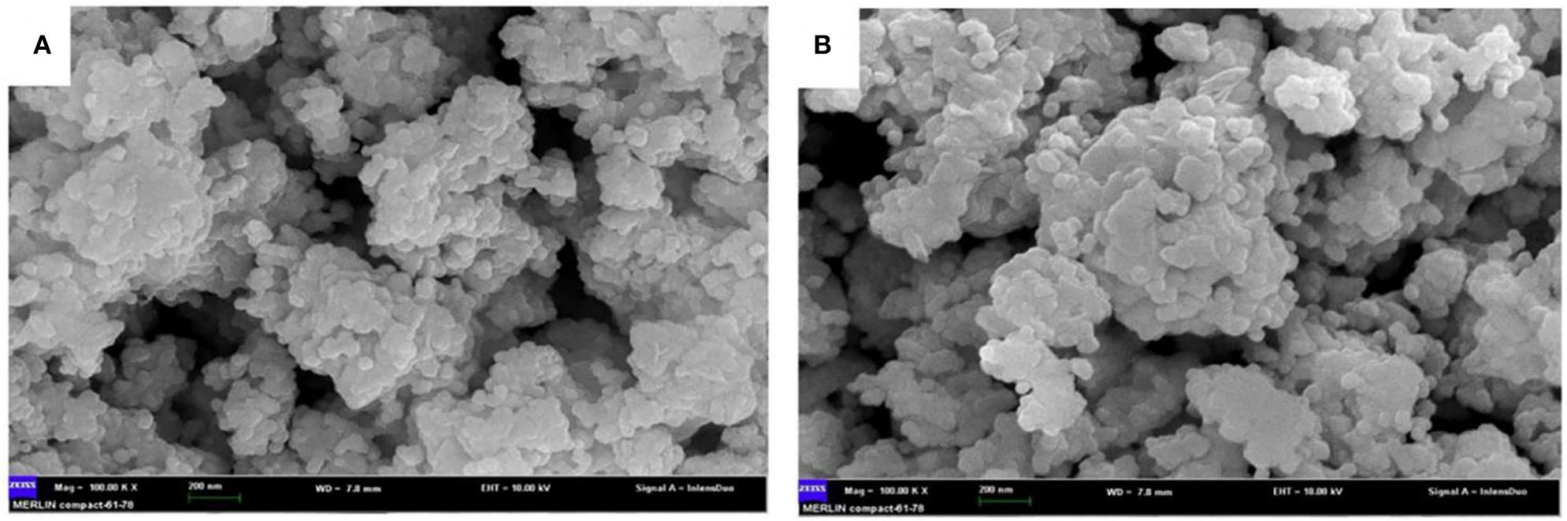
SEM images of **(A)** Li_2_ZnTi_3_O_8_, **(B)** Li_2_ZnTi_2.9_Cr_0.1_O_8_.

Figure 3 shows the SEM images of the Li_2_ZnTi_3_O_8_ and Li_2_ZnTi_2.9_Cr_0.1_O_8_ samples. It can be clearly seen that both samples are well-crystallized, with a small grain size distribution. It should be noted that the morphology of the particles did not appreciably change following doping with minute amounts of Cr^3+^. While increasing the contact area between active particles and the electrolyte, a good dispersion can narrow the transmission distance between Li^+^ and the electrons and increase the high-rate performance of 5C.

**Figure 4:**
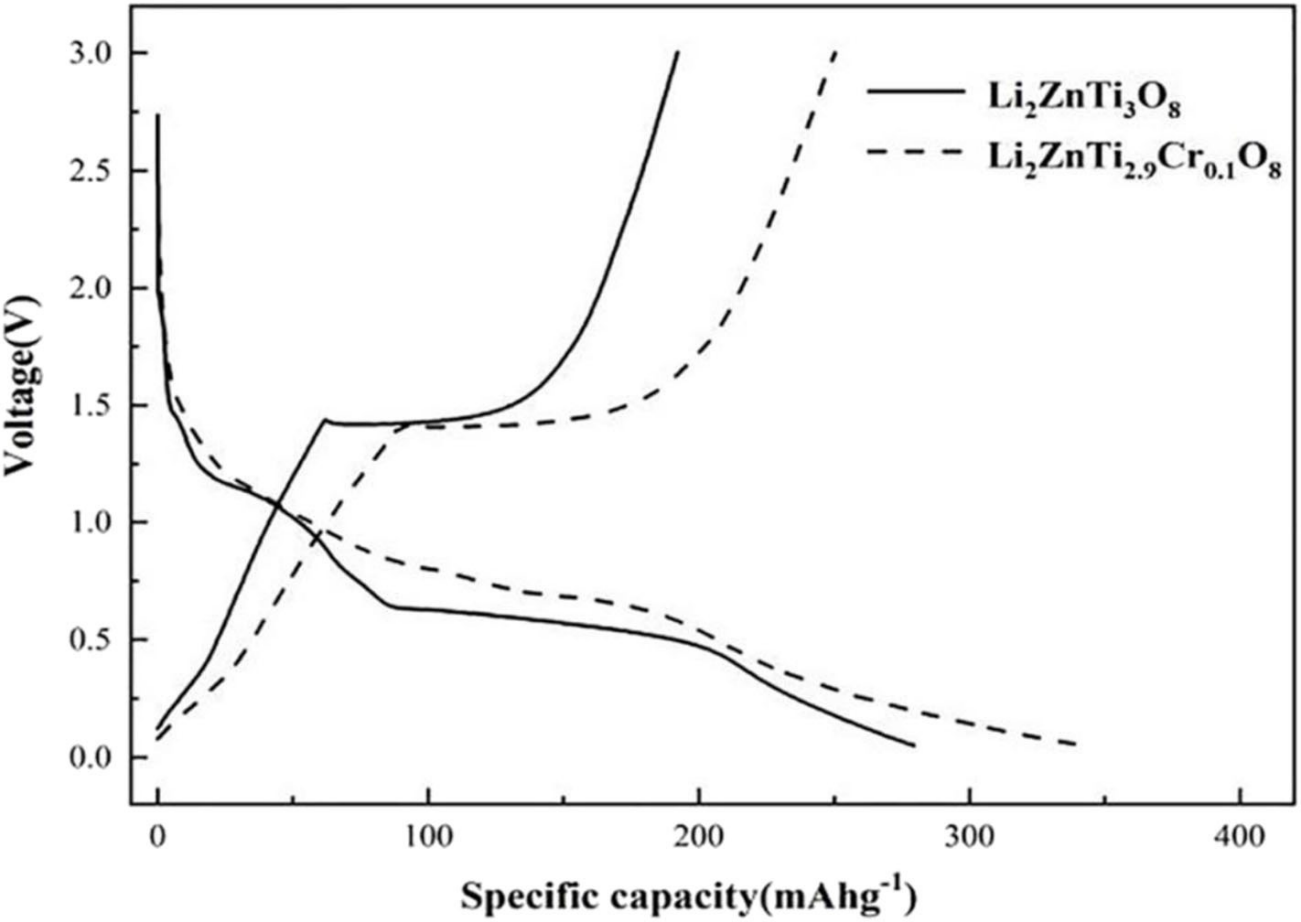
The 1st charge and discharge curves for Li_2_ZnTi_3_O_8_ and Li_2_ZnTi_2.9_Cr_0.1_O_8_.

The initial charge/discharge curves of Li_2_ZnTi_3_O_8_ and Li_2_ZnTi_2.9_Cr_0.1_O_8_ are shown in Figure 4, which shows that the charge/discharge curves of Li_2_ZnTi_2.9_Cr_0.1_O_8_ are similar to those of Li_2_ZnTi_3_O_8_, suggesting that Cr^3+^ doping exerts an effect on the electrochemical reaction. The specific capacities of Li_2_ZnTi_3_O_8_ and Li_2_ZnTi_2.9_Cr_0.1_O_8_ at rates of 1 A g^−1^ after the first cycle were 130.5 and 166.8 mA h g^−1^, respectively. As can clearly be seen, Li_2_ZnTi_2.9_Cr_0.1_O_8_ demonstrates a higher specific capacity than Li_2_ZnTi_3_O_8_. This can be explained by the fact that Cr doping can enlarge the transport tunnel of lithium ions, which further increases lithium ion transportation and electron transfer, thus enhancing the electronic conductivity (Zhang et al., 2018). In addition, Li_2_ZnTi_2.9_Cr_0.1_O_8_ appears to have the least voltage platform difference, indicating that a lower electrode polarization was obtained following Cr^3+^ doping and Li^+^ transport was improved.

**Figure 5:**
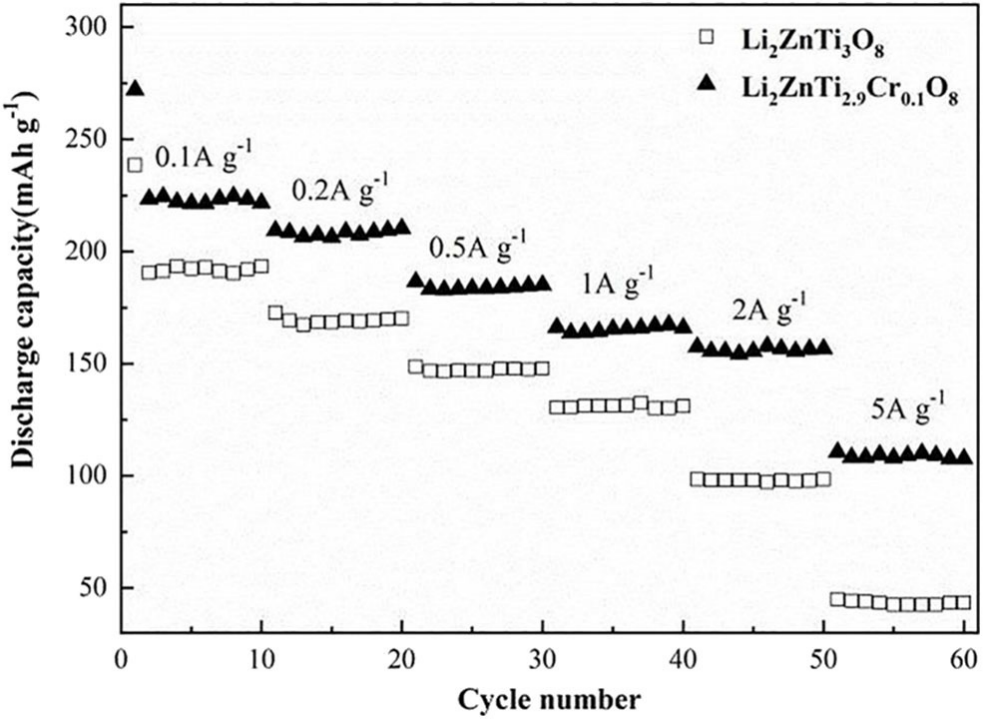
Rate performance at different current density of Li_2_ZnTi_3_O_8_ and Li_2_ZnTi_2.9_Cr_0.1_O_8_.

The rate performances of Li_2_ZnTi_3_O_8_ and Li_2_ZnTi_2.9_Cr_0.1_O_8_ are compared in Figure 5. Li_2_ZnTi_2.9_Cr_0.1_O_8_ delivered maximum discharge capacities of 221.7, 210.3, 185.1, 166, 156.7, and 107.5 mA h g^−1^ at 0.1, 0.2, 0.5, 1, 2, and 5 C, respectively, whereas Li_2_ZnTi_3_O_8_ delivered 193.4, 170.2, 147.8, 131, 98.3, and 43.5 mA h g^−1^ at the same rates, respectively. As can be seen, Li_2_ZnTi_2.9_Cr_0.1_O_8_ showed better rate performance compared with Li_2_ZnTi_3_O_8_, which may be due to either or both of the following reasons: (1) Cr doping improves the electronic conductivity, which leads to Li_2_ZnTi_2.9_Cr_0.1_O_8_ demonstrating better electrochemical performance than Li_2_ZnTi_3_O_8_; (2) the improved cell volume following Cr doping enhances lithium ion diffusion and electron transfer (Chen et al., 2009; Nie et al., 2020).

**Figure 6:**
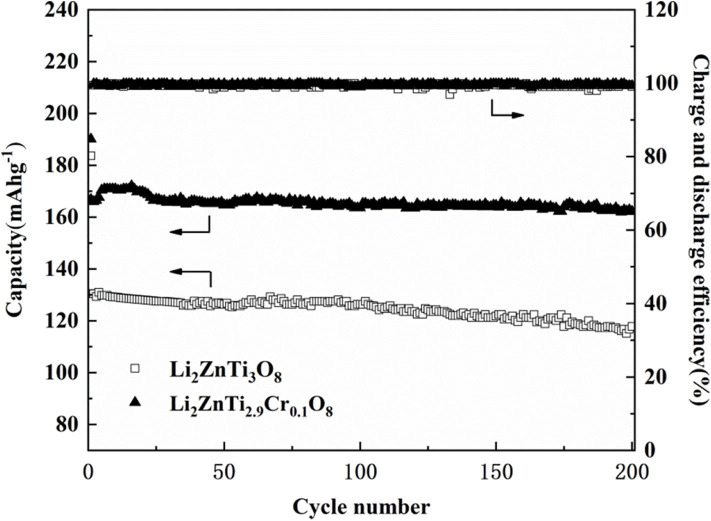
Cycling capacity of Li_2_ZnTi_3_O_8_ and Li_2_ZnTi_2.9_Cr_0.1_O_8_ for 200 cycles.

The cycling performance of Li_2_ZnTi_3_O_8_ and Li_2_ZnTi_2.9_Cr_0.1_O_8_ was examined at a rate of 1 A g^−1^, and the results are shown in Figure 6. As seen, the coulombic efficiency of Li_2_ZnTi_3_O_8_ and Li_2_ZnTi_2.9_Cr_0.1_O_8_ were 80.2 and 84.8% in the first cycle, indicating that the coulombic efficiency can be enhanced after Cr doped. After several cycles, the coulombic efficiency of two samples is close to 100%. After 200 cycles, the reserving ratios were 90.2 and 97.2% for Li_2_ZnTi_3_O_8_ and Li_2_ZnTi_2.9_Cr_0.1_O_8_, respectively. Therefore, Li_2_ZnTi_2.9_Cr_0.1_O_8_ possesses the better cycling performance. These results indicate that Li_2_ZnTi_2.9_Cr_0.1_O_8_ shows better capacity retention than Li_2_ZnTi_3_O_8_, which can probably be explained by assuming that moderate Cr doping can enlarge the transport tunnel of lithium ions, which further increases lithium ion transportation and electron transfer.

**Figure 7:**
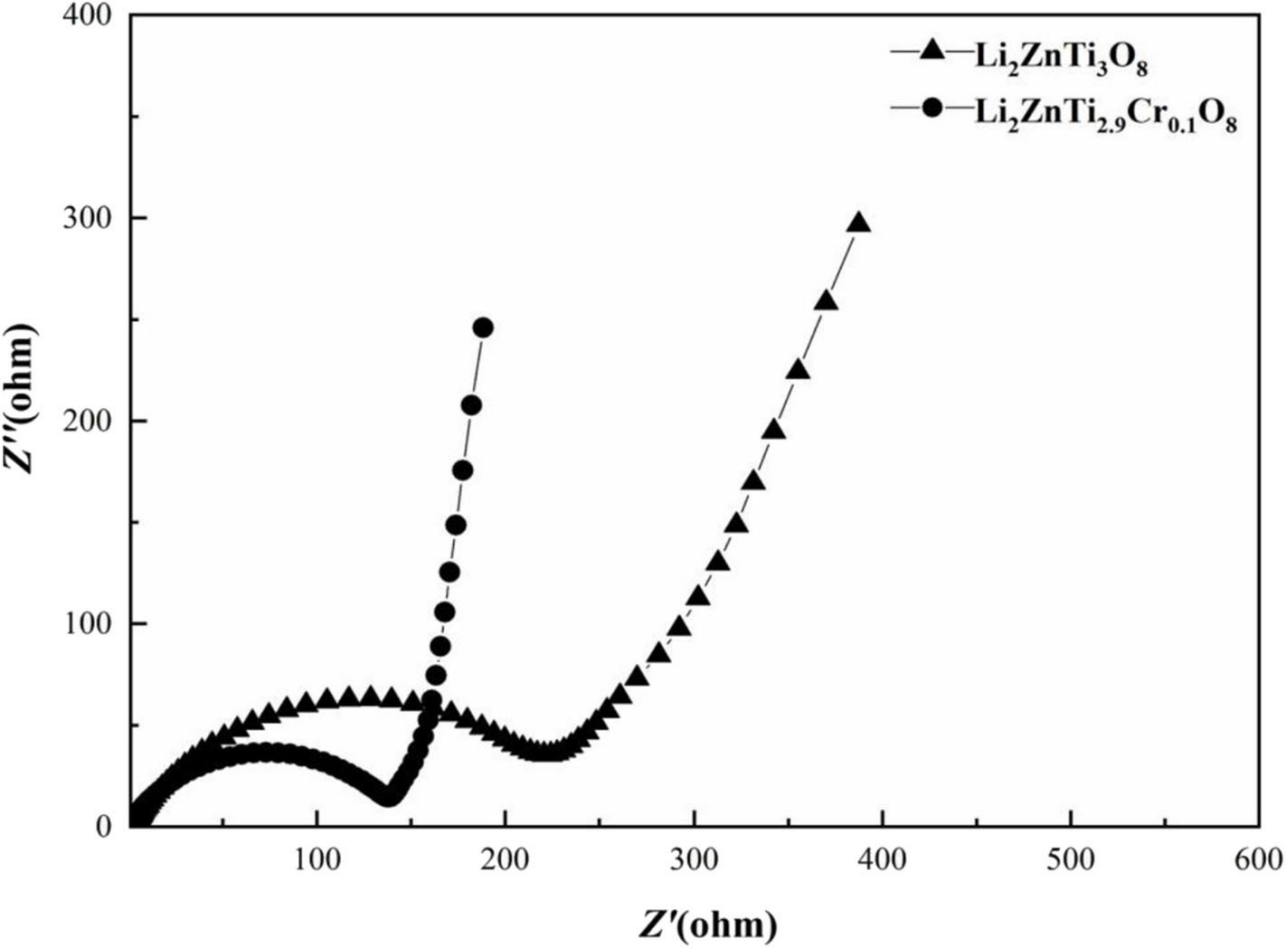
EIS of pure Li_2_ZnTi_3_O_8_ and Li_2_ZnTi_2.9_Cr_0.1_O_8_.

Electrochemical impedance spectroscopy (EIS) measurements for Li_2_ZnTi_3_O_8_ and Li_2_ZnTi_2.9_Cr_0.1_O_8_ were carried out to probe the kinetic properties of these anode materials, and the results are shown in Figure 7. Similar patterns are displayed by the impedance spectra of the two samples, which formed a semicircle in the high-frequency regions and a straight line in the low-frequency regions. The semicircle in the high-frequency regions is associated with charge transfer resistance on the electrode/electrolyte interface, while the straight line in the low-frequency regions is ascribed to the diffusion of Li^+^ into the Warburg resistance (Long et al., 2011; Tang et al., 2019), which constitutes the bulk of the electrode materials. It is clear that the charge transfer resistance of the Li_2_ZnTi_2.9_Cr_0.1_O_8_ electrode material is lower than that of Li_2_ZnTi_3_O_8_, showing that a certain small amount of Cr^3+^ doping of Li_2_ZnTi_3_O_8_ is useful for enhancing the electronic conductivity (Li Z. F. et al., 2016; Kou et al., 2020). In addition, the slope for the Li_2_ZnTi_2.9_Cr_0.1_O_8_ electrode material in the low-frequency regions is slightly higher than that for Li_2_ZnTi_3_O_8_ because Cr doping can strengthen lithium ion migration through Li_2_ZnTi_3_O_8_.

## Conclusions

This study successfully prepared Li_2_ZnTi_3_O_8_ and Li_2_ZnTi_2.9_Cr_0.1_O_8_ samples with the cubic spinel structure by the sol-gel method. XRD revealed that the element Cr successfully doped into the Li_2_ZnTi_3_O_8_ exerted no effect on the spinel structure of Li_2_ZnTi_3_O_8_. SEM (Chen B. K. et al., 2015) results demonstrated that the morphology of the particles did not obviously change following Cr^3+^ doping. The measured electrochemical properties indicated that Li_2_ZnTi_2.9_Cr_0.1_O_8_ shows a better rate performance and excellent cyclic reversibility than Li_2_ZnTi_3_O_8_. The electrochemical performance might be significantly enhanced as a result of the higher electronic conductivity following Cr^3+^ doping, demonstrating that Li_2_ZnTi_2.9_Cr_0.1_O_8_ is a promising anode material for high-rate LIBs.

## Data Availability Statement

All datasets presented in this study are included in the article/supplementary material.

## Author Contributions

XZ and JP contributed conception and design of the study. HZ and YG organized the database. JP and XH wrote the first draft of the manuscript. XZ revised the whole manuscript. All authors contributed to the article and approved the submitted version.

## Conflict of Interest

HZ was employed by the company Langxingda Technology Co, Ltd. The remaining authors declare that the research was conducted in the absence of any commercial or financial relationships that could be construed as a potential conflict of interest.
